# Early perinatal outcomes of babies born to adolescent mothers at two maternity hospitals in Mogadishu, Somalia

**DOI:** 10.4314/ahs.v23i2.82

**Published:** 2023-06

**Authors:** Fartun Orey, Grace Ndeezi, Jamiir Mugalu, Luul Mohamud, Nicolette Nabukeera-Barungi

**Affiliations:** 1 Makerere University College of Health Sciences, Paediatrics and Child Health; 2 Mulago National Referral Hospital, Paediatrics and Child Health; 3 Jazeera University, Department of Paediatrics

**Keywords:** Adolescent mother, early perinatal outcome, poor perinata

## Abstract

**Background:**

Adolescent motherhood remains a major problem in developing countries. We set out to describe the perinatal outcomes of infants born to adolescent mothers and to determine factors associated with birth asphyxia among these infants in Mogadishu, Somalia.

**Methods:**

This cross-sectional study involved adolescent mothers who presented in labor. Sociodemographic and medical data was collected and mother-infant pairs were followed up at 24hrs. Poor perinatal outcomes were: low birth weight, birth asphyxia, death or hospitalization after 24 hours. Data was entered into Epi data 3.1 and analysed using STATA version 12.0.

**Results:**

Of the 270 adolescents, mean age was 17.7 years (±1.19), 89% were married, 94% were unemployed and 54% had not received formal education. Of the 270 babies born, 70 (25.9%) had poor perinatal outcomes which included; 35 (12.9%) died; of whom 30 (11.1%) were stillbirths. Thirty-two infants (13.3%) had birth asphyxia and 18 (6.7%) had low birth weight. Prolonged labor (p-value=<0.001) and preterm birth (p-value=0.023) were significantly associated with birth asphyxia while living in Mogadishu was protective (p- value=0.018).

**Conclusions:**

About one in four adolescent mother's babies had poor perinatal outcomes. Prolonged labor and preterm delivery and were associated with birth asphyxia while residing closer to the facilities was protective.

## Background

According to the World Health Organization (WHO), an adolescent is defined as any person aged 10-19 years^1^. Worldwide, 11% of all births occur among adolescent girls aged 15 to 19 years. Ninety-five percent of these births occur in poorly resourced countries 2. In Somalia, where adolescents contribute about 57% of the population and early marriages are culturally acceptable 3, the burden of adolescent motherhood and their perinatal outcomes are unknown.

Several factors predispose adolescents to poor perinatal outcomes. Adolescent mothers are still young and therefore not well developed both psychologically and physiologically 4. They are also more likely to be socially and economically disadvantaged, which could limit their access to adequate nutrition and health care services 4. Therefore, they are likely to experience pregnancy related complications such as malnutrition given their high nutritional requirements, which in turn affect their unborn babies 5. Moreover, maternal weight before pregnancy and during pregnancy impacts on perinatal outcomes such as preterm births and intra uterine growth retardation (IUGR) 5. In comparison to older mothers; adolescent mothers are more likely to be less educated and unmarried 4. Adolescent mothers are less likely to have received early prenatal care, more likely to have a low career aspiration, residence in a single parent home, poor family relationship as well as no availability/non utilization of contraceptive services 4, 6, 7. The combined effect of low socioeconomic status and high nutritional needs predispose to complications in both antenatal and intra- partum periods 6-9. Adolescent mothers are more likely to have babies with birth asphyxia because they are more likely to be primigravida, have cephalo-pelvic disproportion, and be delivered by caesarean section 10.

Birth asphyxia is described as a failure to initiate and sustain default breathing at birth 11. It is the third leading cause of mortality in neonates globally 10. In developing countries, nearly 3% of about 120 million new born babies develop asphyxia each year 10 and approximately 90,000 neonates die due to birth asphyxia 12, 13. Adolescent mothers are more prone to have babies with birth asphyxia, because they are more likely to experience prolonged and obstructed labor due cephalo-pelvic disproportion 13.

In addition, the civil war in Somalia has led to collapse of health and educational systems and this has led to school non-attendance and drop-out among girls. This combined with poverty contributes to early marriage resulting into teenage pregnancies. Furthermore, early marriage is culturally accepted in Somalia since it is believed that a girl can get married as long as she has attained menarche. This further promotes adolescent pregnancy with far reaching implications on the adolescent girl-child and her unborn baby.

In 2015, the neonatal mortality rate in Somalia was at 40 per 1000 live births, contributing to nearly a third of under-five mortality. Conditions contributing to such a high mortality rate in Somalia include birth asphyxia, prematurity, sepsis and LBW 14 which have been reported to be worse among adolescent mothers 15. Findings of this study will be used as a basis for future research and provide evidence-based knowledge about perinatal problems of babies born to adolescent mothers. Results of the study will also be used to increase public awareness of perinatal problems associated with early marriage in the Somali population, further contributing to the reduction in neonatal morbidity and mortality in the country.

This study therefore, aimed to describe the early perinatal outcomes of babies born to adolescent mothers at SOS and Benadir hospitals. And also, to determine the factors associated with birth asphyxia among adolescent mothers at SOS and Benadir hospitals.

## Methodology

### Study design and setting

This study was prospective cross-sectional study conducted between July 2016 and December 2016 in the maternity and neonatal wards in Benadir and SOS maternity Hospitals in Somalia. Both hospitals are public, referral and teaching hospitals located in the capital city of Somalia -Mogadishu.

### Sampling

We included adolescent mothers who presented in labor and delivered at the two selected hospitals. We also included all new born babies whose adolescent mothers consented to participate in the study. A total of 270 young mothers 10 to19 years were enrolled into the study

### Study procedure

Prior to starting the study, all midwives in the hospital and research assistants were trained in new born resuscitation by the principal investigator (PI). For each hospital, two research assistants were recruited who included one doctor and one midwife. The research assistants (one doctor and one midwife from each hospital) (RA) were trained to do the Ballard score to determine the gestational age of the new born baby using new Ballard score. Data collection took place daily. The screening was done at the labor suite to determine eligibility.

Adolescent mothers were identified using the eligibility criteria at admission. Eligible adolescent mothers were taken through a verbal consent process by the PI/RA at admission. The RA or PI did not intervene until after delivery when written informed consent was sought. After consent, the study questionnaire was administered by the PI/ RA while they were in the postnatal wards. The delivery data was extracted from the hospital file. For mothers who were admitted and delivered at night, written informed consent was sought in the morning and then the parts of the questionnaire's concerning delivery was completed.

Questionnaires were used to capture information about maternal factors such as the age of the mother, mode of delivery, parity, duration of labor, marital status, occupation, residence, level of education, maternal health (hypertension, diabetic mellitus), drug or medicine use and abuse. The neonatal characteristics were obtained soon after birth and at 24 hours of life or at discharge.

The weight of the mother was taken after delivery by the PI or RA. The midwife who delivered the mother or who received the baby after Caesarean section observed and recorded the Apgar score at 1 and 5 minutes and weighed the baby. The midwife or the doctor who delivered the baby considered any new born baby who had no signs of life at birth as stillbirth. Later the weight of the baby, stillbirth, Apgar score and mode of delivery were extracted from the babies' hospital files by the PI/RA. The PI or RA assessed the new Ballard score and performed the general exam of the baby looking for any obvious dysmorphic features in the first twelve hours. Any mother who needed urgent care was immediately sent to the doctor on duty for management and study procedures were stopped and only continued when the mother was stable. For all mothers, the questionnaire was administered after birth and was completed at discharge or at 24 hours after delivery to assess the outcome. The neonates who had poor perinatal outcome were admitted to the neonatal unit and their final condition was determined at 24 hours after birth. All neonates who were stable received the standard of care which included Vitamin K, Tetracycline eye ointment and immunizations.

### Measuring weight of the mother

The weight measurement was taken after delivery following Rolland S. Gibson's (1993) guidelines (16). The weighing scales (SECCA) were calibrated to zero before each measurement. The RA assigned to a particular study site took the weight. The weight reading was then read off from the scale after stabilization. Two readings were obtained to ensure accuracy and the average taken as the final value. The weight was recorded to nearest 100gms.

### Measuring height of the mother

The mothers' height was taken using a height tape pinned to the wall and reading recorded to the nearest centimetre. Measurements were repeated once to ensure accuracy and the average of the two values taken as the final value. BMI was calculated as *WT(kg)/HT(cm)*^2^.

### Ethical Considerations

Ethical approval was obtained from the Makerere University College of Health Sciences, School of Medicine Research and Ethics committee *REC REF 2016-072 and the Uganda National Council for Science and Technology and Ministry of Health of Somalia.

Institutional consent to conduct the study was sought from the management of both Benadir and SOS maternity Hospitals and informed consent was obtained from the mothers. The consent form was translated into the Somali language. It was agreed by the institutional review board in Uganda and MOH in Somalia that although some of these mothers were below 18years, a written informed consent for involvement in the study should be obtained from them since they were regarded as emancipated minors. The mothers were informed that their participation in the study was voluntary and they were free to withdraw from the study at any time without any consequences.

### Data analysis

Univariable data was analysed through descriptive statistics such as frequency and percentage. Bivariate chi square analysis with crude odd ratio with 95% confidence interval were conducted with categorical variables Factors with a p-vale less than 0.2 were considered for multivariable analysis. Associations with a p-value ≤ 0.05 were considered statistically significant after adjusting for interaction and confounding only those who were significant on multivariate shown on the table.

## Results Demographics

A total of 314 pregnant adolescents who presented in labor were screened for this study, 33 declined while 7 of them were very ill to participate and 4 lost follow up. Of these, 270 adolescent mothers participated in the study. The average age of the mothers was 17.7 (±1.2) years and 26.7 (±6.3) years for the fathers, with 89% (242/270) of the mothers married. This study found that 54% (146/270) of the adolescent mothers had no formal education, 86.3% (233/270) were residents of Mogadishu and 94% (256/270) were housewives. Most mothers lived within 5 kilometers from the hospital and were regarded as having easy accesshile those who lived in a distance of more than 20km were regarded as having poor access. Other characteristics are summarized in table 1and 2 below.

**Table 1:** Socio demographic characteristics of adolescents who delivered from Benadir and SOS N=270

Variables	Frequency (N=270)	Percentage (%)
**Age of mother (yrs)**		
≤15	14	5.19
16-17	88	32.59
18-19	168	62.22
**Age of father (yrs)**		
16-25	144	53.33
26-35	100	37.04
≥36	26	9.63
**Parity**		
Prim Gravid	229	84.81
Multigravida	41	15.19
**Marital status of the mother**		
Married	242	89.63
Divorced	25	9.26
Widow	3	1.11
**Level of education**		
Non-formal education	146	54.07
Primary	69	25.56
Elementary	30	11.11
Secondary and above	25	9.26
**Residence**		
Mogadishu	233	86.30
Other	37	13.70
**Distance to hospital (km)**		
<5	205	75.93
5-10	26	9.63
10-20	11	4.07
>20	28	10.37
**Occupation of mother**		
House wife	256	94.81
Other	14	5.19
**Weight of the mother (kg)**		
40-50	65	24.07
51-60	134	49.63
>60	71	26.30
**Height of the mother (cm)**		
<100	17	6.30
100-160	130	48.15
>160	123	45.56
**BMI of the mother**		
<18	16	5.93
18-25	204	75.56
>25	50	18.52

**Table 2:** Maternal characteristics of enrolled adolescent mothers who delivered babies at Benadir and SOS hospital N=270

Variables	Frequency(N) =270	Percentage (%)
**Antenatal care attendance (visit)**		
No	80	29.64
<4	148	54.81
≥4	42	15.56
		
**Took prophylaxis against malaria**		
Yes	89	32.96
No	181	67.04
**Took iron and folic supplementation**		
Yes	178	65.93
No	92	34.07
**Suffered any febrile illness during pregnancy**		
Yes	76	28.15
No	194	71.85
Use any medication during pregnancy*		
Yes	98	36.30
No	172	63.70
**Use any herbal medicine while pregnant**		
Yes	9	3.33
No	261	96.67
**Hypertension**	17	6.30
**Diabetics**	1	0.37
**Tuberculosis**	3	1.11
**Checked Haemoglobin during pregnancy**		
Yes	119	44.07
No	151	55.93
**Duration of labour (Hrs)**		
<24	175	64.81
>24	95	35.19
**Mode of delivery**		
Spontaneous vaginal delivery	205	75.93
Caesarean	34	12.59
Forceps	26	9.63
Vacuum	5	1.85

### The early perinatal outcomes of babies born to adolescent mothers

About 25.9% (70/270) of babies had poor perinatal outcomes. This included prematurity, LBW, admission to the neonatal ward or ICU due to other causes, and mortality within 24 hours of birth. Those born with low birth weight (<2.5kg) were 18 (6.7%). Among the low-birth-weight babies, preterm delivery contributed to 72.2% (13/18) and 3 babies were extremely pre-term (<28 weeks), 6 were very preterm (28 to <32 weeks) and 4 moderates to late preterm (32 to <37 weeks).

The proportion of babies who died was 35/270 (12.9%), of which 11.1% (30/270) died at birth and 2 % (5 babies) died within 24 hours. The 35 deaths out of 270 births implies 130 deaths per 1000 live births in this hospital-based population among adolescent mothers. This is over 3 times the national level.

Babies who had congenital anomalies or trauma were 3.3% (8/240). Sixty-seven babies were not discharged at 24 hours and the main reason was low Apgar score (22/67 babies). Other reasons for hospital stay for more than 24 hours were being born by caesarean section or maternal illness. Perinatal outcomes of the babies are summarized in Table 3.

**Table 3:** Early perinatal outcomes of the babies born to adolescent mothers N=270

Variables	Frequency (N) = 270	Percentage (%)
**Perinatal outcomes**		
Poor Outcome	70	25.93
**Gestational age (weeks)**		
Preterm (24-36)	13	4.81
Full term ≥37	257	95.19
**Baby alive at birth**		
Yes	240	88.89
No	30	11.11
Apgar Score at 5 minutes*		
<7	32	13.33
≥**7**	208	86.67
Birth Weight (kg)*		
<2.5	18	6.67
≥2.5	252	93.33
Baby had birth trauma*		
Yes	6	2.50
No	234	97.50
Baby had dysmorphic feature*		
Yes	2	0.83
No	238	99.17
Baby alive after 24 hrs*		
Yes	235	97.92
No	5	2.08
The baby discharged within 24 hrs*		
Yes	168	71.49
No	67	28.51
Reason for no discharge within 24hrs (67) *		
Caesarean section	13	19.7
Birth asphyxia	22	33.33
Mother was sick	13	19.7
Small for gestational age	4	6.06
Others	15	21.21

### Factors associated with birth asphyxia among babies of adolescent mothers

The prevalence of birth asphyxia among the babies was 13.3% (32/240).

At multivariable analysis, gestational period, duration of labor and residence were significantly associated with birth asphyxia as shown in the table 4 and table 5

**Table 4:** Bivariate and multivariate analysis of the demographic factors associated with birth asphyxia among babies born to adolescent mothers. N=240

Variables	No birth asphyxia (n) =208	Birth Asphyxia (n)= 32	COR (95% CI)	AOR (95% CI)
**Age of mother (Yrs)**				
≤15	10(71.4)	4(28.6)	1	1
16-17	64(85.3)	11(14.7)	0.43(0.11-1.62)	0.393(0.09-1.71)
≥18	134(88.7)	17(11.3)	0.32(0.09-1.12)	0.270 (0.07-1.11)
**Age of father (Yrs)**				
16-25	115(88.5)	15(11.5)	1	
26-35	72(82.8)	15(17.2)	1.60(0.74-3.46)	---------
≥36	21(91.3)	2(8.7)	0.73(0.16-3.43)	
Parity				
Prime Gravida	175(85.8)	29(14.2)	1	
Multigravida	33(91.7)	3(8.3)	0.55(0.16-1.91)	
**Gestational age (weeks)**				
Preterm (24-36)	2(50.0)	2(50.0)	1	1
≥37	206(87.3)	30(12.7)	0.15(0.02-1.07) *	0.060 (0.01-0.68) *
**Marital status of the mother**				
Married	188(87.0)	28(13.0)	1	1
Divorced/widowed	20(83.3)	4(16.7)	1.34(0.42-4.21)	1.281 (0.34-4.84)
**Level of education**				
Non-formal education	111(86.7)	17(13.3)	1	
Primary	50(82.0)	11(18.0)	1.44(0.63-3.29)	---------
Elementary	24(85.7)	4(14.3)	1.09(0.34-3.52)	
Secondary and above	23(100.0)	0(0)		
**Residence**				
Mogadishu	190(88.8)	24(11.2)	0.28(0.11-0.72) **	0.277 (0.10-0.79) **
Other	18(69.2)	8(30.8)	1	1
**Distance to hospital (Km)**				
<5	165(88.2)	22(11.8)	1	
5 and above	43(81.1)	10(18.9)	1.74(0.77-3.96)	---------
**Occupation of mother**				
House wife	198(87.2)	29(12.8)	0.49(0.13-1.88)	---------
Other	10(76.9)	3(23.1)	1	
**Weight of the mother (Kg)**				
40-50	50(92.6)	4(7.4)	1	
51-60	101(82.8)	21(17.2)	2.60(0.85-7.98)	---------
>60	57(89.1)	7(10.9)	1.54(0.42-5.55)	
**Height of the mother (Cm)**				
<160	98(47.12)	11(34.38)	1	
≥160	110(52.88)	21(65.63)	1.70(0.78-3.71)	---------
**BMI of the mother**				
Underweight	8(80.0)	2(20.0)	1	
Normal weight	160(87.0)	24(13.04)	0.56(0.12-2.99)	---------
Overweight	40(86.96)	6(13.04)	0.56(0.10-3.53)	

*** P-value < 0.001

** P-value < 0.01

* P-value < 0.05

AOR adjusted Odds Ratio, COR Cruds Odds Ratio CI Confidence Interval

**Table 5:** Bivariate and multivariate analysis for maternal and neonatal factors associated with birth asphyxia. N=240

Variables	No birth asphyxia	Birth Asphyxia	COR (95% CI)	AOR (95% CI)
**Antenatal care attendance (visit)**				
No	58(85.3)	10(14.7)	1	
<4	113(85.6)	19(14.4)	0.98(0.42-2.23)	----
≥4	37(92.5)	3(7.5)	0.47(0.12-1.82)	
**Reasons for not attending at least 4 ANC visits**				
Not aware	140(85.9)	23(14.1)	0.49(0.15-1.66)	----
Busy	11(91.7)	1(8.3)	0.27(0.03-2.83)	
Lives far from MCH	8(88.9)	1(11.1)	0.38(0.04-4.00)	
Others	12(75.0)	4(25.0)	1	
**Took prophylaxis against malaria**				
Yes	71(88.8)	9(11.2)	0.76(0.33-1.72)	----
No	137(85.6)	23(14.4)	1	
**Took iron and folic supplementation**				
Yes	141(88.1)	19(11.9)	0.69(0.32-1.49)	----
No	67(83.8)	13(16.2)	1	
**Duration of labor**				
<24hrs	149(93.7)	10(6.3)	1	1
≥ 24hrs	59(72.8)	22(27.2)	5.56(2.48-12.43) ***	6.852 (2.84-16.50) ***
**Mode of delivery**				
Spontaneous vaginal delivery	168(89.8)	19(10.2)	1	
Caesarean	21(77.8)	6(22.2)	2.53(0.91-7.03)	
Forceps	17(70.8)	7(29.2)	3.64(1.33-9.90) **	
Vacuum	2(100.0)	0(0.0)	-	
**Suffered any febrile illness during pregnancy**				
Yes	59(88.1)	8(11.9)	0.84(0.36-1.98)	----
No	149(86.1)	24(13.9)	1	
**Use any medication during pregnancy**				
Yes	78(91.8)	7(8.2)	0.47(0.19-1.12)	0.403 (0.15-1.10)
No	130(83.4)	25(16.1)	1	1
**Checked Hb during pregnancy**				
Yes	94(86.2)	15(13.8)	1.07(0.51-2.26)	1.836 (0.75-4.51)
No	114(87.0)	17(12.9)	1	1
**Birth Weight (Kg)**				
<2.5	6(66.7)	3(33.3)	3.49(0.83-14.69)	----
≥2.5	202(87.4)	29(12.6)	1	
**Baby has dysmorphic feature or trauma**				
Yes	3(37.5)	5(62.5)	12.6(2.86-55.95) ***	
No	205(88.4)	27(11.6)	1	

*** P-value < 0.001

** P-value < 0.01

* P-value < 0.05

AOR adjusted Odds Ratio, COR Cruds Odds Ratio CI Confidence Interval

Babies with at least 37 weeks of gestation were associated with 94% reduced odds of having birth asphyxia compared to those with gestational age less than 37 weeks (preterm) (OR:0.060, p=0.023). Mothers from Mogadishu were 72% less likely to have babies with birth asphyxia compared to mothers from other parts of the country (OR: 0.277, p=0.018).

Mothers who had prolonged labor (duration of labor ≥ 24 hours) were 6.9 times more likely to have babies with birth asphyxia compared to mothers who did not have prolonged labour (OR: 6.852, p=<0.001).

## Discussion

### Early perinatal outcomes of babies born to adolescent mothers

Results of this study indicate that about 26% of the recruited babies had abnormal early perinatal outcomes. These included birth asphyxias, prematurity, LBW, admission to the neonatal ward or ICU due to other causes, and mortality within 24 hours of birth. Poor perinatal outcomes among adolescent mothers have been known to be associated with poor physical and psychological immaturity, increasing the risk of getting babies with; birth asphyxia, LBW, and/or stillbirth 4. This is also confounded by findings of this study that showed that majority of the adolescent mothers had poor education and were not financially independent Dr. Rupakala B M et al in India reported findings similar to ours with 29.4% of adolescent mothers studied having abnormal fetal outcomes 17. In Uganda, Nabirye Loy, in a non-published study reported that 15% of the enrolled adolescent mothers had poor perinatal outcomes which is relatively lower than what we found in the current study 18.

The difference in prevalence between the current study and Loy's could be due to the higher ANC attendance reported in the Ugandan study.

### Low birth weight

Result of this study showed that 6.67% of the babies had low birth weight. Similar result (8.7%), were reported in a Population- based multi-country research among adolescent mothers (15-19 years) from sub-Saharan Africa and Latin America 2. This means that proceeding in Somalia is similar to what is happening in some of the other sub-Saharan African countries.

In a study conducted in Thailand 19 LBW rate was reported to be 10.4%, higher than what is reported the current study. The higher percentage in the study conducted in Thailand could have been that it was a comparative study, comparing outcomes of pregnancies in both adolescent and adult mothers and their study also had a larger sample size of 1068 participants. adolescent have poor ANC visits so that some complications like infections (malaria, syphilis) and hypertension that could be ascertained during ANC visits may be missed and probably contribute to LBW depicted in the various studies alluded to above.

### Babies who died in 24 hours

Our study found that 12.9% of babies died of which most were stillbirths (11.1%). Stillbirth rate is much higher in this study compared to other studies done in Nigeria and Indiay Garba et al and Rupakala et al of 2.5% and 1.04% respectively 17, 20. The factors contributing to the high mortality in this study compared to the other studies are poor ANC care attendance, low socioeconomic status, low educational level and lack of awareness about ANC care and perinatal screening. In addition, the higher mortality in the current study could be explained by the fact that Somalia is recovering from a protracted civil war, which has led to very poor health care delivery in the country. However, in the current study, 2% of babies died within 24 hours. There was no other study in the similar duration of follow up to compare with. However, an Indian study reported that 5.1% of babies of adolescent mothers died in 48 hours. The higher percentage of mortality in the Indian study could have been due to the long duration of follow up 21. Overall, this indicates that mortality neonatal mortality of infants of adolescent mothers is higher than in the older mothers. It is reported that there are 50% higher stillbirths and newborn deaths among infants born to adolescent mothers than among those born to mothers aged 20–29 years 22.

### Prolonged duration of hospital stays

In this study, 28.5% of the babies were still admitted by the end of follow up period. The commonest (33%) cause of hospital admission was birth asphyxia. This is because the children are still sick and need attention in hospital. Our study is in line with findings from other studies done in India and Eritria by Rupakala et al and Shah et al respectively 23-24. The two studies reported that birth asphyxia is one of the commonest causes of prolonged hospital stay. This is because adolescent mothers are prone to have prolonged labor or obstructed labor due to cephalo-pelvic disproportion, which leads to a prolonged hospital stay.

### Factors associated with asphyxia among adolescent mothers

In the current study, 13.33% of babies had birth asphyxia. A similar study done by Mukhopadhyay et al reported a slightly higher prevalence of 16.6% amongst adolescent mothers in India 21. The study was comparative in design looking at teenage primigravida mothers and adult primigravida mothers. This is because primigravida mothers are more likely to have complications like obstructed labor and likely to deliver babies with birth asphyxia. In the current study, the percentage of primigravida was 84.8%. The current study reported that being born a preterm was strongly associated with developing birth asphyxia. In a similar study conducted in Nepal, Lee et al reported similar results 25.

In the current study, prolonged labor was significantly associated with birth asphyxia. Similar findings have been reported in Nepal and Brazil 25, 26. As discussed earlier, adolescent mothers are not mature physically to handle pregnancy due to their narrow pelvis. They are at increased risk of Cephalo-pelvic disproportion leading to a prolonged labor. The situation is worse among primigravida. r Our study had a very high figure of 84.8% being first-time mothers, explaining the high rate of birth asphyxia.

Mothers from Mogadishu (an urban area), were less likely to have babies with birth asphyxia compared to mothers from other parts of the country. This is because mothers in Mogadishu are nearer to the hospital and can access it easily. Mothers who stay far from Mogadishu are more likely to experience more than one delay like delay reaching health care, delayed referral or delay to receive care, which is problematic in case of complications like prolonged labor, which eventually leads to birth asphyxia. So, taking these mothers to a service that is closer to them may result in better outcomes.

Being married was not significantly associated with poor outcomes in this study, probably because nearly 90% of adolescent mothers were married. This is similar to a study done by Presler-Marshall & Jones, where they found 90% of adolescent births in developing countries were from married girls and the majority of adolescent pregnancies were actually planned 27. In Somalia, early marriage is culturally acceptable. In addition, poverty and poor school enrolment may contribute to early marriage in girls below^18^ years of age.

The strength of this study is that to our knowledge, this is the first study undertaken to assess early perinatal outcomes in adolescent mothers in Somalia. This is a rampant problem which has not been addressed. This study is very helpful in advocacy against early marriages and teenage pregnancies at a national level.

The studies limitations were; First, we were not able to get the 1st trimester ultrasound scan so we relied on maternal LMP and Ballard score to estimate the gestational age.

Secondly, since this study was cross-sectional with a short duration of follow up, we could not ascertain the outcome of the babies after 24 hours.

Thirdly, we did not find out the effect of FGM on perinatal outcomes yet the practice is common in Somali communities and has a potential of affecting perinatal outcomes.

## Conclusions

One in four adolescent mothers had poor perinatal outcomes. A mother having prolonged labor or a baby being born preterm was significantly associated with birth asphyxia.

However, being a resident of Mogadishu, an urban area was protective.

## Recommendations

Since most adolescent mothers in this study were married and had non-formal education but generally with poor perinatal outcomes, efforts to delay early marriage and pregnancy should be done at all levels by supporting girl-child education. In addition, the government should put into place laws on defilement and should be intentional in preventing early marriages. Alleviating poverty by financial support to help families and opportunities for decent work after school will also keep the girl child in school and reduces early marriage. Lastly, the Government should improve access to specialised health care and promote health education so that adolescent mothers learn the importance of attending Antenatal care and delivery by skilled health workers.
